# INTERGENERATIONAL COMMUNITY DEVELOPMENT: LESSONS LEARNED FROM THE SENIOR TO SENIOR DINNER DANCE PROGRAM

**DOI:** 10.1093/geroni/igae098.2934

**Published:** 2024-12-31

**Authors:** Youjung Lee, Jacqueline McGinley

**Affiliations:** Binghamton University, Binghamton, New York, United States; Binghamton University, Binghamton, New York, United States

## Abstract

Intergenerational engagement is a universal human development process that helps older adults’ successful aging (Krzeczkowska et al., 2021) and children’s optimal development (McAlister et al., 2019). As part of an age-friendly initiative in a small city in New York State, an intergenerational engagement program (Senior to Senior Dinner Dance) involved a multi-year partnership between community agencies, including a local Office for Aging, university, high school, senior center, and church. As part of this partnership, a community dance was held in 2022 and 2023, where high school students and older adults participated in a variety of intergenerational activities. Findings from a process evaluation of this interorganizational initiative (e.g., focus groups with six community partners; surveys with 56 older adults), using a thematic analysis (Braun & Clarke, 2006), revealed: (a) social cohesion included mutual trust amongst community stakeholders; (b) social capital was achieved by leveraging existing resources with collective goals; and (c) intergenerational engagement was promoted through the equal status, common goals, and interorganizational cooperation of participants, in addition to sanctions and support from organizers. Findings from this study highlight important lessons for those, particularly those in communities with limited resources, who wish to develop and sustain intergenerational programs. Lessons learned include the importance of mutual trust among community partners, the utilization of existing resources, and the necessity of buy-in from upper administration in each agency. Lastly, intergenerational activities (e.g., dining, dancing) can promote initial positive contact between distinct age cohorts, which may help to advance larger age-friendly initiatives.

